# Hepatocellular Carcinoma in Pakistan: Where do We Stand?

**DOI:** 10.5812/hepatmon.6023

**Published:** 2012-10-10

**Authors:** Amna Subhan Butt, Zaigham Abbas, Wasim Jafri

**Affiliations:** 1 Section of Gastroenterology, Department of Medicine, The Aga Khan University Hospital, Karachi, Pakistan

**Keywords:** Carcinoma, Hepatocellular, Pakistan, Hepatitis C

## Abstract

**Context:**

From the 1970s till the mid 1990s, hepatitis B was the most common etiological factor for hepatocellular carcinoma (HCC) in Pakistan. Afterwards, a shift in HCC etiology was observed with a steady rise in hepatitis C virus (HCV) related HCC cases. HCV-3a, which is the most prevalent genotype, is also most frequent in HCV related HCC. There was an increase in the proportion of non-B non-C (NBNC) HCC cases as well, which might be attributed to an increase in non-alcoholic fatty liver disease.

**Evidence Acquisition:**

The age-standardized rate for HCC is 7.64/100 000 in males and 2.8/100 000 in females. Male to female ratio is 3.6:1. Usual age of presentation is in the fifth and sixth decade. Most patients present with advanced disease, as they are not in a regular surveillance program. This is more so for patients with NBNC chronic liver disease. As many sonologists in Pakistan are practicing without sufficient training to pick up early lesions, alpha-fetoprotein is still recommended to compliment ultrasound in the surveillance of HCC.

**Results:**

Majority of HCC patients present with nonresectable disease. Interventions such as transarterial chemoembolization, radiofrequency ablation, resection and chemotherapy including sorafenib are available in selected centers. Pakistan appears to be in an area of intermediate endemicity for HCC. There is a need for population based epidemiological studies to estimate the exact disease burden.

**Conclusions:**

Measures to prevent the spread of hepatitis C and B can slow down the epidemic rise in the incidence of HCC in the coming decades. There is a need to implement a proper surveillance program to identify HCC cases at an early stage.

## 1. Context

Hepatocellular carcinoma (HCC) is the sixth most common cancer globally, attributing to 626 000 or 5.7% of new cancer cases annually (1). It is the third most common cause of cancer related deaths globally and carries an overall survival rate of only 3-5%. The major burden of HCC lies in developing countries; up to 82% of HCC cases are reported from developing countries, including 55% from China alone (1-3). Moreover, relatively high incidence rates have been found in South East Asia and in Sub-Saharan Africa (2-4). Hepatitis B and C are the major risk factors for HCC (5). Geographical variations in the significance of hepatitis B virus (HBV) and hepatitis C virus (HCV) infections in the development of HCC, clinical features and survival have been reported (1, 6, 7). However, the evidence regarding risk estimates for the development of HCC in individuals coinfected with HBV, HCV and hepatitis D (HDV) is lacking (3).

Pakistan is located in South-Asia where the prevalence of hepatitis B and C is intermediate (6, 7). Considering the burden of hepatitis B and C, it is expected that the incidence of HCC will increase further in the foreseen future, especially in countries such as China and Taiwan (8). Hence, HCC will be a major burden on their healthcare systems (3). This review is based on evidence gathered from published data related to the; magnitude, etiological factors, disease characteristics, therapeutic response and survival of HCC patients in Pakistan.

## 2. Evidence Acquisition 

### 2.1. Search Strategy

Medline indexed database, Google Scholar and a local search engine, i.e. PakMediNet (http://www.pakmedinet.com), which also lists local studies that are not listed in Medline) as well as abstracts from major international hepatology conferences, were used to search for relevant articles published during January 1970 till September 2011. Mesh terms; “Hepatocellular Carcinoma and Pakistan”, “Hepatoma and Pakistan”, “Primary liver cancer and Pakistan”, “HCC and Pakistan” and “liver cancer and Pakistan” were used for the search. In addition, relevant studies were identified by reviewing the reference lists of selected articles. Those articles that were relevant and where their full text article or complete abstract was available and the sample size was at least 20, were included in this review.

## 3. Result

### 3.1. Epidemiology

Unfortunately, no population based study was available from which a true prevalence and incidence rate of HCC could be ascertained. Most of the studies were hospital based, consisting of case series with small sample sizes or they had a highly select population. However, there have been a few cancer registries established in Pakistan. The Karachi Cancer Registry (KCR) was the first population-based cancer registry, established in 1995, by the Sindh Government, in technical collaboration with the Unit of Descriptive Epidemiology, International Agency for Research on Cancer (IARC) of the World Health Organization (WHO) (9). The Aga Khan University Cancer Surveillance for Pakistan (ACSP) was established in 2000 at the Aga Khan University Pathology-based Cancer Registry (APCR), which covers a large geographical area and population of Pakistan, through their 84 centers. Moreover, APCR is an associate member of the IACR (10). The incidence and prevalence of the various cancers has been estimated in certain cities via these registries. Out of 4 268 new cancer cases registered in the KCR from Karachi, district South during 1995-1997, the age-standardized rates (ASR) for HCC were found to be 5.7/100 000 in males and 3.7/100 000 in females (rank 9^th^) (9). Furthermore, ASR (world population was taken as a reference here) were found to be 5.3 and 4.0/100 000 in Karachi during 1998 to 2002 (11). Moreover, among the cancers registered to KCR and APCR from Larkana during 2000-2002, ASR for HCC were 10.5/100 000 persons in male and 2.0/100 000 in females (12). Likewise, cancer patients who were residents of Hyderabad and who were registered in KCR and APCR during 1998 to 2002, their ASR for HCC were reported to be 4.4/100 000 in males and 1.2/100 000 in females (10). From Quetta, 1 077 cancers were registered in KCR during 1998 to 1999. Here, HCC was found to be the third most common cancer in men (age standardized rate 12.3/100 000) and the seventh most common cancer in women (ASR 3.1/100 000) (13). Hence, ASR reported an average figure in these different studies as 7.64/100 000 for males and 2.8/100 000 for females. Another hospital based tumor registry has been established at the Armed Forces Institute of Pathology (AFIP). Out of 21 168 cancers reviewed during 1992-2001, liver cancers were found in 22 (2.81%) males and 22 (2.81%) females (14).

**Figure 1.**
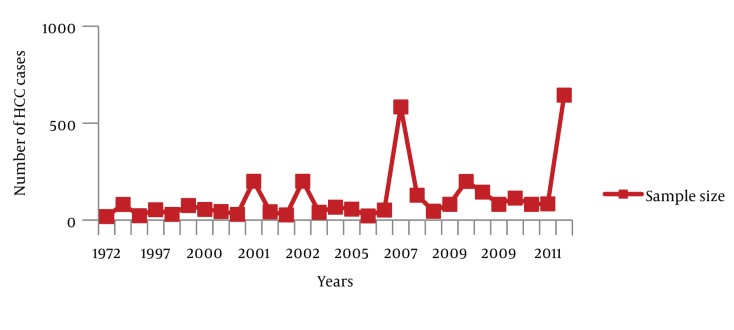
Distribution of HCC Cases, 1970-2011

**Figure 2.**
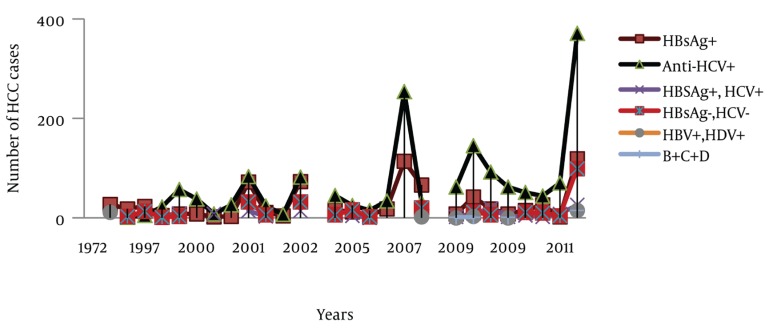
Comparison of Hepatitis B and C Related HCC and NBNC-HCC During 1970-2011

**Figure 3.**
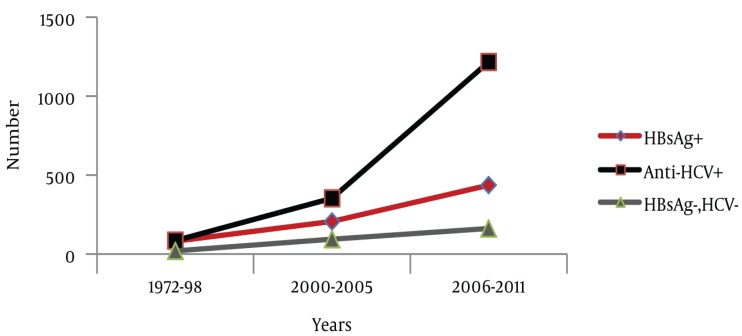
Comparison of Various Etiological Factors for HCC, 1970-2011

**Figure 4.**
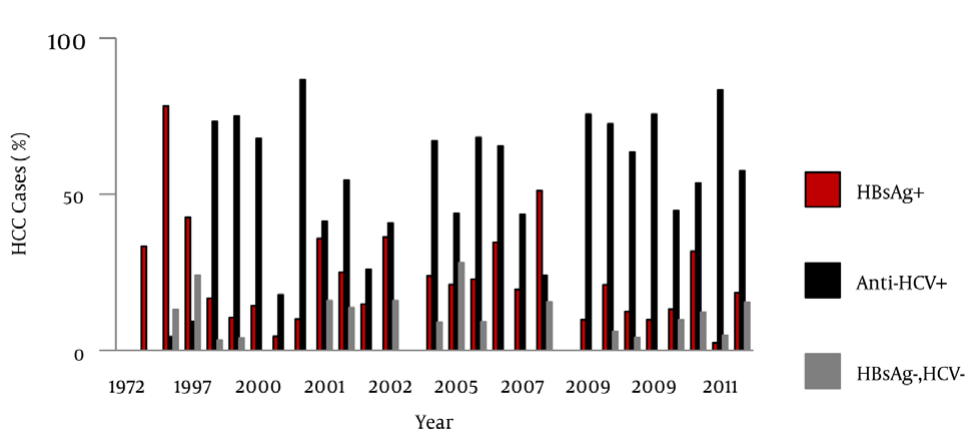
Comparison of Hepatitis B and C related HCC and NBNC-HCC during Different Time Intervals

Besides these registered data, 29 relevant studies were found where the epidemiological aspects of HCC were evaluated (Table 1). The numbers of cases from these 29 studies were aggregated (n = 3319) and trends of HCC, gender distribution and etiological factors were analyzed. Out of the 3 319 cases, information regarding gender was available for 2 967 cases. Out of 2 967 cases, 78.29% were male and 21.71% were female. Age ranges were between 8-98 years. However, in most of the studies HCC cases presented during the fifth decade of life. Information about the etiological factors for HCC was available in 2 852 cases; HCV, HBV and HCV/HBV co-infection was found in 57.99%, 25.35% and 5.26% cases respectively. Coinfection with HBV and HDV was found in 1.09% cases and hepatitis B/C/D co-infection was found in 0.63% cases. Moreover, 9.68% cases were seronegative for hepatitis B and C (NBNC-HCC). As it is clear from Figure 1, the number of studies related to HCC increased with the passage of time. Hence, a rise in the number of HCC cases was observed during the study period. During the entire study, an almost consistent male preponderance was found in the HCC cases. As far as the etiology of HCC is concerned, it has been identified that from the 1970s till the mid 1990s, hepatitis B was the most common etiological factor for HCC. Afterwards, a shift in HCC etiology with a steady rise in HCV related HCC cases were observed (Figure 2). Moreover, an inconsistent rise in the number of HCC cases attributed to HBV/HCV or HCV/HBV/HDV co-infection and NBNC-HCC was also recognized (Figure 3). To evaluate the data further, we divided the time period into three categories; 1. 1970-1999 2. 2000-2005 and 3. 2006-2011, and the data was compared for these different time periods (Table 2). Again a clear, consistent shift was recognized in the etiology of HCC (Figure 4). Replacement of HBV by HCV as the major etiological factor for HCC was quite clear. Moreover, a rising trend was seen in the number of NBNC-HCC cases during these time periods. Hence, viral hepatitis is identified as the major attributable factor of HCC in Pakistan. Hepatitis C followed by hepatitis B, HBV/HCV coinfection and HBV/HDV coinfection are the foremost factors leading to HCC. Considering the increasing prevalence of diabetes, hypertension and dyslipidemia in Pakistan, we can assume that the rising numbers of NBNC-HCC might be attributed to non-alcoholic fatty liver disease (NAFLD) which requires further studies for confirmation. Furthermore, in Pakistan, a high rate of food contamination with aflatoxin has been reported earlier, and this is a known carcinogen for HCC in humans (15, 16). However, there is a lack of data regarding aflatoxins leading to HCC from Pakistan.

**Table 1.** Age, Gender Distribution and Etiological Factors for HCC in Pakistan, 1970-2011

	Study Period	Sample Size	Location	Male, No. (%)	Female, No. (%)	Age Mean±, (range)	HBsAg+, No. (%)	Anti-HCV+, No. (%)	HBsAg+AntiHCV+, No. (%)	HBsAg-ve, anti HCV-ve, No. (%)	HBV+, HDV +, No. (%)	B+C+D
**Shah M*, et al.* (1972) (39)**	1963-1971	19	Peshawar	12 (63.2)	6 (36.8)	10-70	-	-	-	-	-	-
**Qureshi H*, et al.* 1990 (40) **	1969-1985	81	Karachi			8-98	27 (33.3)	-	-	-	11 (40.7)	-
**Tong CY*, et al.* 1996 (41)**	1987-1992	23	Rawalpindi	22 (95.6)	1 (4.4)	47 (2-76)	18 (78.3)	1 (4.3)	1 (4.3)	3 (13.0)	-	-
**Abdul Mujeeb S*, et al.* 1997 (42)**	1997	54	Karachi	-	-	-	23 (42.6)	5 (9.3)	13 (24.1)	13 (24.1)	-	-
** Kausar S*, et al.* 1998 (43)**	1998	30	Lahore	-	-	-	5 (16.7)	22 (73.3)	2 (6.7)	1 (3.3)	-	-
**Butt AK*, et al.* 1998 (21)**	1998	76	Lahore	65 (85.5)	11 (4.5)	52.2 ± 11.3	8 (10.5)	57 (75.0)	8 (10.5)	3 (3.9)	-	-
**Farooqi JI*, et al.* 2000 (44)**	1995-1998	56	Peshawar	JCPSP			8 (14.28)	38 (67.8)	-	-	-	-
**Farooqi JI*, et al.* 2000 (45)**		45	Peshawar	JCPSP			2 (4.44)	8 (17.78)	8 (17.78)	-	-	-
**Chohan AR*, et al.* 2001 (18)**	2000	30	Rawalpindi	20 (66.7)	10 (33.3)	59.2	3 (10)	26 (86.6)	-	-	-	-
**Sharieff S*, et al.* 2001 (46)**	1994-198	201	Karachi	149	52	-	72 (35.8)	83 (41.3)	14 (7.0)	32 (15.9)	-	-
**Mumtaz MS*, et al.* 2001 (47)**	2000	44	Rawalpindi	-	-	-	11 (25)	24 (54.5)	3 (6.8)	6 (13.6)	-	-
**Khokhar N*, et al.* 2001 (22)**	1995-99	27	Islamabad	19	8	3 5-84	4 (15)	7 (26)	-	-	-	-
**Sharieff S*, et al.* 2002 (28), **	1994-1998	201	Karachi	149 (74)	52 (16)	56 (24-85)	73 (36)	82 (41)	14 (7)	32 (16)	-	-
**Khokhar N*, et al.* 2002 (48)**	1994-2000	41	Islamabad	-	-	-	(14)	(29.3)	-	(53)	-	-
**Khokar N*, et al.* 2003 (24)**	1994-2000	67	Islamabad	53	14	58.64 ± 12.77	16 (23)	45 (67)	-	6 (9)	-	-
**Hamza H*, et al. *(49), 2005**	2005	57	-	40	17	-	12 (21.1)	25 (43.9)	4 (7.0)	16 (28.1)	-	-
**Gill ML*, et al.* 2005 (36)**	2003	22	Islamabad	22	00	52.5 (45-60)	5 (23)	15 (68)	00	2 (9)	-	-
**Ziauddin*, et al.* 2006 (50)**	2004-2006	52	Peshawar	45 (86.5)	7 (13.5)	(12-100)	18 (32)	34 (68)	-	-	-	-
**Yusuf MA*, et al.* 2007 (26), **	1995-2004	584	Lahore	444	140	56	114 (19.5)	254(43.5)	-	-	-	-
**Abbas Z*, et al. *2008 (35)**	2002-2006	129	Karachi	97 (75)	32 (25)	52 (18 – 82)	66 (51.2)	31 (24 ),	10 (7.8),	20 (15.4)	02 (1.6),	-
**Baig JA*, et al. * (51) **	2004-2005	46	Karachi	39	7	23-60	-	-	-	-	-	-
**Khan A*, et al*. 2009 (17)**	2006-2007	82	Karachi	63	19	55.8 ± 9.9	8 (9.7)	62 (75.6)	2 (2.4)	-	00	1 (1.2)
**Ansari S,* et al*. (23)** **Alcohol +HCV = 22**	2005-2008	200	Hyderabad, Sindh	165 (82.5)	35(17.5)	53.7 ± 12 (20-90)	42 (21.0)	145 (72.5)	9 (4.5)	12 (6.0)	3 (1.5)	1 (0.5)
**Idrees M*, et al*. 2009 (25) a**												
**2001-2009**	145	Punjab and NWFP	107 (73.7)	38 (26.3)	58 ± 11	18 (12.41)	92 (63.4)	19 (13.10)	6 (4.13)	-	-	
**Khan A,* et al*. 2009 (17)**	2006-2007	82	Karachi	63	19	55.8±9.9	8 (9.7)	62 (75.6)	2 (2.4)	-	0	1(1.2)
**Ali R, *et al*. 2010 (19)**	2005-2009	114	Islamabad	85	29	56.4 ± 10.25	15 (13.1)	51 (44.7)	5 (4.3)	11 (9.6)	-	-
**Abbasi A,* et al*. 2010 (20)**	2008	82	Karachi	58	24	56.24 ± 13.65	26 (31.7)	44 (53.7)	2 (2.44)	10 (12.2)	-	-
**Nawaz AA, *et al*. 2011 (52) b**	2008-2011	84	Punjab	60 (72)	24 (28)	80% > 50 years of age	2 (2)	70 (83)	8 (9)	4 (5)	-	-
**Butt AS*, et al*. 2011 (27)**	1999-2011	645	Karachi	546	99	56.93 ± 11.15 (18-95)	119	371	26	99	15	15

^a^Two patients were found to be tissue-positive by PCR but no anti-HCV antibodies were present, Twenty-eight cases were caused by HBV, of whom 18 (19.31%) also had markers for current HBV infection (HBsAgpositive), and two patients (1.37%) had markers for past infection (HBsAg-negative; anti-HBs Ag positive; anti-HBc positive).

^b^One third (38%) were overweight [BMI > 25]. 26% also suffered from Diabetes Mellitus. 74 [86%] of the patients had liver cirrhosis [Child’s B 51%, Child’s C 35%], 7% ALD.

**Table 2.** HCC Cases during Different Time Periods

Year	Sample Size	Male	Female	HBsAg+	Anti-HCV+	HBSAg + & HCV+	HBsAg- & HCV-	HBV+ & HDV+	B & C & D+
**1970-1999**	283	99	18	81	85	24	20	11	0
**2000-2005**	791	452	153	206	353	43	94	0	0
**2006-2011**	2245	1772	473	436	1216	83	162	20	18

### 3.2. HCV Genotypes and HCC

Hepatitis C is the commonest cause of HCC in most of the developing countries and the distribution of HCV genotypes varies across the world. Hence, studies have been conducted in different populations worldwide to evaluate the association of HCV genotype 1 with HCC development. In Pakistan, the most prevalent HCV genotype is 3. Until now, only two studies have been conducted in Pakistan to study the association of HCV genotypes with HCC. Khan A et al. (17) evaluated 189 patients with chronic liver disease including 82 with HCC. Hepatitis C genotype 3a was the predominant genotype (81.4%) followed by 3b (9.3%), 3k (2.3%), 1a (1.5%), 1c (1.5%), 1b (0.8%), and 2a (0.8%). Out of 82 HCC cases, 76% were infected with genotype 3a. However, considering the small sample size this study lacks the strength to correlate other HCV genotypes with HCC in our population. The additional information that was provided by the study from Khan et al. (17), is the existence of a distinct phylogenetic cluster of genotype 3a in Pakistan, and its appearance in this region in 1920s had a rapid exponential growth in the 1950s. Hence, these findings suggest an earlier epidemic spread of HCV-3a in Pakistan than in the other countries where genotype 3 has been reported. This might be associated with the increasing incidence of HCC in Pakistan during the last few decades. Likewise, in another study the distribution of the HCV genotype found was; 3a in 40.96%, 3b in 15.66%, 1a in 9.63%, and 1b in 2.40% and mixed genotypes in 28.91% of HCC patients. Of the 24 mixed genotypes, ten were infected with genotypes 3a and 3b, eight with 1a and 3a, and six with 1a and 3b. While, two tissue samples were found to be untypable as no genotype was detected (37).

### 3.3. Clinical Presentations of HCC

Most of the available studies have emphasized the epidemiological aspects. In general most of the patients remained; asymptomatic or experienced right hypochondrial pain, weight loss, jaundice, fever, upper gastrointestinal bleed, hepatic encephalopathy, hepatosplenomegaly, abdominal/liver mass and ascites (18-23). Cirrhosis was reported in 69-84% cases with Child’s class B or C in most of the cases (18, 21-25). Out of 400 HCC cases reviewed by Yusuf et al. (26) 216, 147 and 37 patients had Child’s class A, B and C respectively. Whilst in another study, out of 645 HCC cases, the majority had Child’s class B (42.3%) or C (45.1%) cirrhosis (27).

Furthermore, it was an alarming finding that 82.9% of the HCC cases in the largest case series of 645 patients were diagnosed when they were symptomatic and 8.8% were diagnosed incidentally. Whereas, only 8.2% (n = 53) of the HCC cases were diagnosed on screening. The duration between diagnosis of a chronic liver disease and HCC was 24.01 ± 38.05 months (range 0-195 months). Moreover, 480 (74.4%) patients experienced at least one complication related to cirrhosis earlier to their index presentation, and that included; ascites (68.5%), portosystemic encephalopathy (34.1%), esophageal varices (53%), upper gastrointestinal bleeding (40.5%), hepatorenal syndrome (22.6%), hepatohydrothorax (9%), hepatopulmonary syndrome (7.8%) and hypersplenism (62.2%). Among all of the patients, 259 (40.2%), 222 (34.4%) and 16 (2.5%) had concomitant diabetes, hypertension or dyslipidemia respectively (27). In some studies HBV-related HCC patients were found to be younger than HCV-related HCC patients (49.7 v/s 56.3 years) (28). Elevated serum bilirubin (100%), alanine aminotransferase (ALT) (42.1%), aspartate aminotransferase (AST) (42.1%), and alkaline phosphatase (100%), were reported in a group of 145 patients (25). Hence, most of the patients presented with an advanced disease stage, as they were not under a regular surveillance program. This was even true for patients with NBNC chronic liver disease leading to HCC (27). In the process of comparing HCC in HBV mono-infection with HBV/HDV co-infection, it was later found to be associated with a smaller liver size and indirect evidence of more severe portal hypertension in an earlier TNM stage (29).

### 3.4. Diagnosis of HCC

#### 3.4.1. Alpha-Fetoprotein

Alpha-fetoprotein (α-fetoprotein, AFP) is a large serum glycoprotein, used as a tumor marker for HCC. The summary of AFP in various studies is given in Table 3. A wide variability in ranges of AFP was found in the various studies. Elevated AFP was reported in 31-100% cases. However, AFP > 200 or > 400 ng/ml was found to be associated with a greater accuracy in detecting HCC. The diagnostic value of AFP for HCC, was evaluated among 100 biopsy proven HCC cases and 100 healthy subjects, who were found to be HBsAg positive on blood screening (30). AFP was found with 72% sensitivity, 89% specificity, 86.7% positive predictive value, and 76.1% negative predictive value to detect HCC (P < 0.001). Likewise in another study (n = 100), AFP was found to be 72% sensitive and, 89% specific to detect HCC (31). However, no correlation was found between the size of the HCC and AFP levels when assessed in 201 patients (r = -0.155; P = 0.129) (32). There is much debate nowadays about the value of AFP in a surveillance program for HCC. What we have observed here in Pakistan is that many sonologists are practicing with insufficient training to pick up early lesions. Hence, alpha-fetoprotein is generally used as a screening tumor marker to compliment ultrasound liver tests for the surveillance of HCC.

**Table 3.** Alpha-Fetoprotein (AFP) Levels in Patients With HCC

	Sample Size	Mean ± SD or Median	Range	Others, ng/ml	Elevated AFP, %
**Butt AK*, et al.* 1998 (21)**	76	142 ± 155	2.7-1470	-	-
**Shah GG*, et al.* 1999 (53)**	32	-	-	-	84.3
**Khokhar N, 2001*, et al.* (22)**	27	-	43-6300	-	
**Chohan Arm*, et al.* 2001 (18)**	30	-	-	-	63.3
**Sharieff S*, et al.* 2001 (32)**	201	17,027 ± 68, 853	-	> 1000: 35%	35
				Normal AFP: 24%	
**Khokar N*, et al.* 2003 (24)**	67	632.09 ± 1332.31	-	-	80
**Gill ML*, et al.* 2005 (36) **	22	15000 ± 1000	-	-	
**Ziauddin*, et al.* 2006 (50) **	52	-	-	< 200:13.5%	100
				200-400:25%	
				> 400;61.5%	
**Yusuf MA*, et al.* 2007 (26)**	442	4198 ± 262 (median)	1-278,560	> 200:70%	-
**Abbas Z*, et al.* 2008 (35)**	129			> 400:37.5%	31
**Baig JA*, et al.* 2009 (51)**	39	421 ± 59	101–2341	-	-
**Ali R*, et al.* 2010 (19)**	114	-	-	< 100:7%	72.8
				< 100 :10.5%	
				≥ 500:5.3%	
**Abbasi AB*, et al.* 2010 (20) **	82	2582.52	2.54- 65609	≥ 400 in 46.34%	-
**Butt AS*, et al.* 2011 (27)**	645	82.0 (median)	0.95-303717	≥ 20:65.7%	67.5
				≥ 200:41.2%	

#### 3.4.2. Radiological Features of HCC

The presence of arterial enhancement, followed by washout of contrast in the portal-venous and delayed phase, are considered to be typical characteristic features of HCC (19, 33).Tumor size, number of lesions, local or distant metastasis are all prognostic factors and these help to make a decision about treatment. A summary of the various studies which describe radiological characteristics of HCC are given in Table 4. The role of biphasic contrast-enhanced helical CT including hepatic arterial phase (HAP) with portal venous phase (PVP) imaging, in the detection and characterization of HCC was evaluated by Yaqoob J et al. in 40 biopsy proven, HCC cases (34). The detection rate for HCC was 85% with HAP imaging (hyperattenuating = 69, hypoattenuating = 3) when compared to 48% with PVP imaging (hyperattenuating = 2, hypoattenuating = 39) (P = 0.008. Moreover, in 7 (17%) cases HCC was visible only in the HAP images.

**Table 4.** Radiological Features of HCC

	Sample Size	Tumor Diameter Mean ±SD, cm	No. of Lesion	Others
**Butt AK*, et al.* 1998 (21)**	76	7.8 x 8.1	Solitary: 54%	> 8cm: 54%
			Multifocal/diffuse : 46%	
**Shah GG*, et al.* 1999 (53)**	32		Diffuse: 38%	> 10 cm: 31.2%
				Right lobe involved predominantly
**Sharieff S*, et al.* 2001 (32)**	201	8.3 ± 4.0	-	> 5cm: 79.49%
**Khokhar N*, et al.* 2001 (22)**	27		Single: 63%	-
			Multiple: 37%	
**Khokar N*, et al.* 2003 (24)**	67	6.6 ± 1.14	Single: 49%	Rt /left lobe involved: 3/7
			Multiple: 51%	
**Yaqoob J*, et al.* 2004 (34)**	40	3.1	Single: 13 cases	Range for tumor size: 0.8 to 14
			Multifocal: 13 cases	
			Dominant mass with satellite lesions: 12	
			Cluster of contiguous nodules: 2	
**Gill ML*, et al.* 2005 (36)**	22	5.0 ± 1.0		
**Ziauddin *, et al.* 2006 (50)**	52	5.41	> 10cm 15.4%	Rt/left/both lobes involved: 44.2%/5.8%/50%
			5.1-10cm -28.8%	
			2-5cm -55.8%	
**Yusuf MA*, et al.* 2007 (26)**	497	8	Solitary: 33%	
			Multifocal: 52%	
**Ansari S*, et al.* 2009 (23)**	200		Single (< 5cm): 30%	PVT: 18%
			Single (> 5cm): 19.5%	
			Multicentric: 68.34%	
**Abbasi AB*, et al.* 2010 (20) **	82		Single: 68.29%	PVT: 24.39%
			Multiple: 26.82%	> 50% liver involved : 51.21%
			Diffuse : 4.87%	
**Butt AS*, et al.* 2011 (27)**	645	5.62 ± 3.67	Solitary: 38.1%	< 5 cm in 55.7%
			Paucifocal: 40.2%	5-10cm in 33%
			Multifocal: 14.9%	> 10cm in 11.3%
			Massive/infiltrative: 6.8%	PVT: 33.5%
				Extrahepatic spread : 13.2%
				Rt/left/both lobes:60%/12.3/27.5

#### 3.4.3. Liver Biopsy

For the diagnosis of HCC, procedures such as; AFP, liver biopsy, triphasic CT scan and ultrasound of the abdomen have been evaluated in various studies. In a study by Yusuf et al. (26) these different diagnostic modalities were evaluated for HCC. Out of 584 patients, fine needle aspiration (FNA) of the liver lesion was done in 71 cases, a core biopsy in 26 and a lipoidal angiography was carried out in 42 patients. A combination of typical radiological findings on an imaging modality and an elevated AFP (> 200 ng/ml) was found in 70 patients. Moreover, a combination of at least two of these modalities, i.e. characteristic findings on a triphasic CT scan, AFP level > 200ng/ml and positive cytology or histology) was reported in 365 patients. FNA under ultrasound guidance was studied in 60 patients with suspected HCC. Mortality was reported in two cases secondary to uncontrollable bleeding after the procedure (21). In another study, out of 114 patients with suspected HCC, 48 (42.1%) patients were found to have biopsy proven HCC. Histologically, well differentiated, moderately differentiated, poorly differentiated, lamellar HCC were reported in 26.4%, 34.5%, 30.9% and 8.2% patients respectively (27).

### 3.5. Staging and Prognostic Factors for Survival

Different staging systems have been used in different studies. In a study of 76 patients 22%, 61% and 17% were found to have Okuda stage I, II and III respectively (20). Abbas Z et al. (35) estimated the survival of 129 HCC patients. Median follow up was 11 months (range 2-36). Three patients were lost to follow-up. Cumulative deaths reported at six months, one year and at two years were; 22 (21.6%), 62 (60.8%) and 93 (92.2%) respectively. On univariate analysis; hepatitis C as etiology, female gender, presence of ascites, splenomegaly, splenic varices, INR > 1.3, total bilirubin > 1.17 mg/dl, direct bilirubin > 0.4 mg/dl, alkaline phosphatase > 169 IU/L, Model for End-Stage Liver Disease (MELD) Score > 12, Child class B & C, multifocal tumor, and transarterial chemoembolization (TACE) were not offered factors found to be associated with poor survival. However, on multivariate analysis, the overall independent determinants of poor survival were; hepatitis C as etiology, female sex and multifocality of tumor (hazard ratios 3.0, 3.0 and 1.9 respectively). Mean survival was 17.2 months for patients who underwent a TACE procedure, as compared to 12.8 months for those who did not receive TACE (P = 0.015). Okuda, Cancer of the Liver Italian Program (CLIP), Barcelona Clinic Liver Cancer (BCLC), Chinese University Prognostic Index (CUPI) and Child’s staging systems retained their performance as judged by chi-square values in a regression analysis. Discriminatory ability for death, evaluated by the receiver operating characteristic curve, was better for the Okuda classification system in the first year.

### 3.6. Treatment and Survival

Unfortunately fewer studies were available regarding the treatment of HCC from Pakistan. In general, data regarding outcomes after curative therapies, i.e. liver transplantation, hepatic resection and radio frequency ablation, is not available. However, scanty data is available concerning the various palliative therapies. In a study by Yusuf et al. (12), only 79 out of 584 patients were found to be eligible for different modalities of treatment. Out of the 79, 48 patients underwent transarterial chemoembolization (TACE) using lipiodol and doxorubicin. Of these, 26 had disease progression, 11 had a stable disease state for a minimum of six months (range 6 - 20 months), and 11 patients were lost to follow-up. Local resection was done in 14 patients and 5 remained disease free for an average of 33 months. Percutaneous ethanol injection (PEI) was performed in 17 patients. Of these, 5 progressed within three months of treatment, 2 had a stable disease state for a mean of 13 months and 10 were lost to follow-up. Four patients underwent TACE followed by surgical resection. The overall median survival was 10.5 months. The cumulative probability of survival was 45%, 20% and 10% at 1-year, 3-years and 5-years. Median survival was better for patients with Child’s class A (12months) when compared to Child’s B (7.7 months) and Child’s C (4.1%). Moreover, the difference in median survival between patients with Child’s class A and B, or A and C was statistically significant (P < 0.001) (26). In the study by Abbas Z et al. (35), 41 HCC patients underwent a TACE procedure. Overall the median survival rate was better for patients offered vs. not offered TACE (14.0 months vs. 9.5 months, OR 1.63, 95% confidence interval 1.07-2.48, P < 0.05). TACE was found to be an effective palliative therapy in another group of 35 patients with unresectable HCC. The median survival time was 410 days. Moreover, a significant difference in mean survival time was found among the different Child’s classes (p-value 0.002) (14). In another study of 201 patients, the median survival reported was only 16 weeks (28). Correlation between AFP level and survival was evaluated further. Survival was 17.6 ± 11.5 weeks for patients with an AFP level less than 10 ng/ml, 17.9 ± 21.7 weeks for patients with AFP levels between 10 to 1 000 ng/ml and 13.2 ± 15.6 weeks for patients with an AFP level > 1 000 ng/ml. However, this difference was not statistically significant. Moreover, no difference in survival rates was found for HBV or HCV related HCC (32). 

Somatostatin receptors have been identified in HCC. Hence, long acting octreotide (LAR) has been tried in the treatment of HCC. In 2003, Gill et al. (36) evaluated the efficacy of LAR in the treatment of inoperable HCC in 22 patients, and the 20 patients who had refused the treatment were used as controls. Patients received 100 mcg octreotide (subcutaneously) thrice daily for two weeks, followed by monthly administration of 20 mg intramuscular octreotide. A total of 19 patients completed the six month treatment. Regression in tumor size and reduction in mean AFP levels were reported in 45.5% and 50% of the cases respectively. Moreover, improvement in the quality of life at the end of treatment was seen in 45.5% cases. At the end of the six month treatment, 64% of the patients were alive in the intervention group as compared to 50% in the control group. In another study conducted by Frooqi et al. (37), 13 advanced HCC cases were randomized to receive either 250 ug subcutaneous octreotide twice daily (6 patients) or no treatment (7 patients). Significant improvement in the quality of life amongst the treatment group was observed, as compared to the control group (P < 0.05). In both studies, the authors recommended octreotide as an alternative for the treatment of inoperable HCC. However, both studies lacked the power to conclude that octreotide had a beneficial effect, due to the small sample sizes and possible selection bias. In a small series of 76 patients, 22 patients received intralesional alcohol injections. However, 54% of these patients with a tumor size > 8 cm died during follow up; this was due to liver failure in 8 cases and fatal bleeding occurred in 4 patients (21). In a study done by Anis et al., oral capecitabine monotherapy failed to halt tumor growth in advanced hepatocellular carcinoma (38). In general, studies regarding the curative treatment of HCC are not available. There are only a few cases that underwent resection of HCC which has been mentioned in several studies. Current existing data regarding treatment and prognosis carries the limitation of small sample size; it is mainly based upon single center experiences and lack of proper follow-ups. Hence, reporting the results of curative and various palliative treatment modalities will provide evidence regarding the outcome and natural history of our HCC cases.

## 4. Conclusion

Hepatitis C related chronic liver disease has become the major cause of a rising prevalence of HCC in Pakistan. The country appears to be in an area of intermediate endemicity for HCC. There is a need for population based epidemiological studies to estimate the exact disease burden. Measures to prevent the spread of hepatitis C and B can slow down the epidemic rise in the incidence in the coming decades. There is a need to implement a proper surveillance program to pick up the disease early. Treatment facilities are not widely available as data about treatment outcomes is scarce. The availability of a sufficient number of patients opens opportunities to do translational research, however, this is lacking at the moment.
